# Deep learning object detection-based early detection of lung cancer

**DOI:** 10.3389/fmed.2025.1567119

**Published:** 2025-04-28

**Authors:** Kuo-Yang Huang, Che-Liang Chung, Jia-Lang Xu

**Affiliations:** ^1^Division of Chest Medicine, Department of Internal Medicine, Changhua Christian Hospital, Changhua, Taiwan; ^2^Institute of Genomics and Bioinformatics, National Chung Hsing University, Taichung, Taiwan; ^3^Ph. D. Program in Medical Biotechnology, National Chung Hsing University, Taichung, Taiwan; ^4^Department of Internal Medicine, Yuanlin Christian Hospital, Changhua, Taiwan; ^5^Department of Computer Science and Information Engineering, Chaoyang University of Technology, Taichung, Taiwan

**Keywords:** lung cancer, early diagnosis, object detection, YOLO, smart medicine

## Abstract

The early diagnosis and accurate classification of lung cancer have a critical impact on clinical treatment and patient survival. The rise of artificial intelligence technology has led to breakthroughs in medical image analysis. The Lung-PET-CT-Dx public dataset was used for the model training and evaluation. The performance of the You Only Look Once (YOLO) series of models in the lung CT image object detection task is compared in terms of algorithms, and different versions of YOLOv5, YOLOv8, YOLOv9, YOLOv10, and YOLOv11 are examined for lung cancer detection and classification. The experimental results indicate that the prediction results of YOLOv8 are better than those of the other YOLO versions, with a precision rate of 90.32% and a recall rate of 84.91%, which proves that the model can effectively assist physicians in lung cancer diagnosis and improve the accuracy of disease localization and identification.

## Introduction

Cancer is one of the deadliest diseases in the world, causing numerous deaths each year (1), and lung cancer is the primary cause of cancer deaths in Taiwan. From 2002 to 2008, there were 33,919 patients with lung cancer (2), and from 2010 to 2016, there were 71,334 patients with lung cancer (3), which increased annually, a phenomenon in which doctors need to pay special attention. In the U.S., there has been a downward trend over the past five years, but it remains the leading cause of cancer-related deaths (4). Lung cancer can be divided into two types: Small Cell Lung Cancer (SCLC) and Non-Small Cell Lung Cancer (NSCLC), which easily metastasizes and is more invasive; therefore, it accounts for 15% of all lung cancers (5). The types of NSCLC are Adenocarcinoma, Squamous Cell Carcinoma (SCC), and Large Cell Carcinoma (LCC). In order to enable physicians to detect lung cancer more quickly, computed tomography (CT) scans are currently employed, as they are more sensitive than chest X-rays and can detect symptoms of early lung cancer earlier (6). Positron Emission Tomography/Computed Tomography (PET/CT) is a whole-body noninvasive imaging technique that allows for more precise evaluation of lymph nodes and metastatic sites (7).

Computer-aided detection (CAD) is a system that can extract features from potential lesions or areas of interest to physicians. However, the system faces the possibility of false positives and negatives (8). Mushtaq et al. (9) suggested that there are two main types of CAD: texture- and non-texture-based methods, while non-texture-based methods usually utilize deep learning for diagnosis. Qin et al. (10) suggested that CAD can help to identify patients with undetected TB, but in high-burden areas, the accuracy of CAD may be reduced due to the presence of previous TB patients. With the emergence of smart healthcare, many scholars are conducting research in the field of healthcare with the addition of artificial intelligence to help healthcare professionals make faster diagnoses, and many scholars are using convolutional neural networks (CNNs) in medical imaging. Lakshmanaprabu et al. (11) proposed the use of Optimal Deep Neural Network (ODNN) and Linear Discriminant Analysis (LDA) for the analysis of thoracic CT image scans. Pang et al. (12) used densely connected convolutional networks (DenseNet) to classify malignant tumor images and then used the adaptive boosting (adaboost) method to aggregate multiple classification results. Wang et al. (13) proposed a new residual neural network model for the classification of CT images and concluded that this method is superior to other algorithms. Gautam et al. (14) used three recent convolutional neural network models: ResNet-152, DenseNet-169, and EfficientNet-B7 to compare and accurately classify the severity of pulmonary nodules.

This study investigated an object detection technique for lung cancer using the YOLO object detection model to train and validate the model and evaluate whether it can effectively detect the types of lung cancer and their locations in CT images. Early diagnosis of lung cancer is crucial for improving patient survival. We developed an efficient Computer-Aided Diagnosis (CAD) system to assist physicians in classifying and localizing lung cancer more accurately. Five different versions of the YOLO model (YOLOv5, YOLOv8, YOLOv9, YOLOv10, and YOLOv11) were selected to evaluate their performances on a CT image dataset for lung cancer. The advantages and limitations of the different versions of the YOLO model were compared through model training and testing, and their accuracy, reasoning speed, and applicability were analyzed.

## Literature review

**Figure 1:**
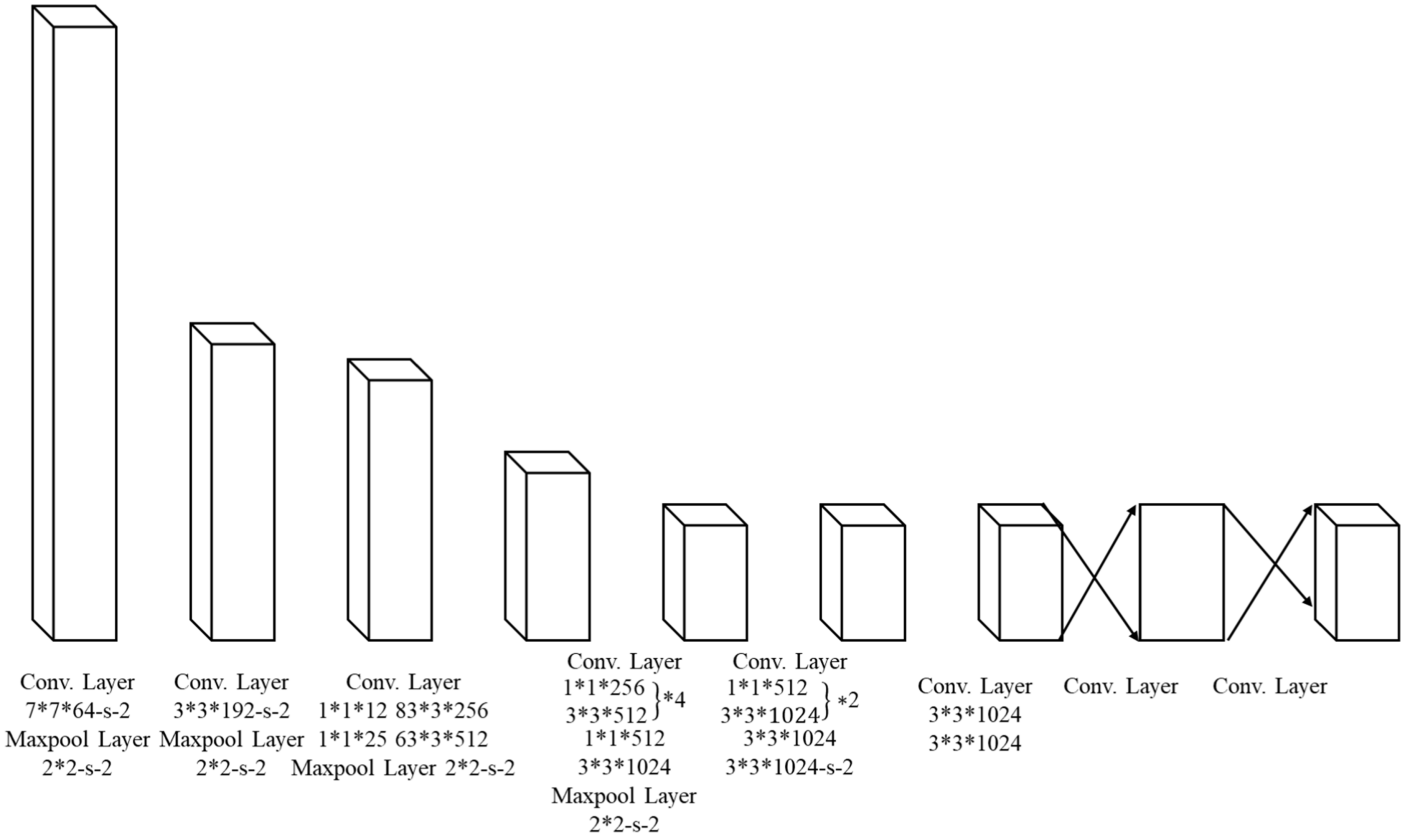
YOLOv1 structure.

Object detection is a core technology used in computer vision. This method can be carried out in films or photos to identify and provide the object’s bounding box and category. Object detection is mainly divided into two modes: Two-Stage Detection and Single-Stage Detection. Two-Stage Detection models include Faster R-CNN, R-FCN, and Cascade R-CNN (15). Faster R-CNN proposed by Ren et al. (16) can perform feature extraction, area proposal network, role pooling, classification, and bounding box regression. The R-FCN model proposed by Dai et al. (17) differs from Faster R-CNN by eliminating RoI Pooling and replacing it with Position-Sensitive Score Maps. The Cascade R-CNN model proposed by Cai and Vasconcelos (18) raises the IoU threshold through multi-stage detectors so that the model can adjust the bounding box more accurately. Single-stage object detection models such as YOLO and Single Shot MultiBox Detector (SSD) are commonly utilized (19). YOLO, proposed by Redmon (20), is a model for image segmentation that uses a mesh (the architecture is shown in Figure 1). SSD was proposed by Liu et al. (21); it uses multi-scale features to detect objects of different sizes.

Figure 1 shows the backbone of YOLOv1, which is a VGG-16-like Convolutional Neural Network (CNN) containing 20 layers of Convolutional Layer (Conv) for extracting image features and 2 layers of Fully Connected Layer (FC) for outputting the prediction results so that the spatial features can be extracted from the image step by step. The image size was also reduced by max-pooling to minimize the computational effort. Finally, a fully connected layer was used to compress the features into a format that can be exported.

## Research model

### Object detection and classification

This study compared five different versions of YOLO to determine whether they could effectively predict the CT images of lung cancer. This study selected YOLOv5, YOLOv8, YOLOv9, YOLOv10, and YOLOv11, which represent different stages of the evolution of the YOLO family, each of which differs in terms of Accuracy, Inference Speed, Architecture Innovation, and Computational Efficiency. This study aims to identify the most suitable version of YOLO for CT image analysis of lung cancer in order to provide more accurate tumor detection and classification, thereby enhancing the reliability and clinical value of medical image diagnosis. The following section provides a detailed description of the five different versions of YOLO, including their core architecture, technical innovations, strengths, limitations, as well as their potential applications in medical image analysis.

### YOLOv5

YOLOv5, proposed by Jocher et al. (22), uses an anchor-free split ultralytic head approach, bypassing the traditional need to define a bounding box to predict the position of an object. The proposed method can effectively calibrate the balance between the speed and accuracy. Begum and Devi (23) proposed a method for brain disease detection using the YOLO framework and concluded that this method can increase the precision of medical image analysis and improve patient care and outcomes. Chen et al. (24) proposed edge tracking of stroke lesions using TE-YOLOv5 and concluded that this method can automatically and effectively detect stroke lesions from DWI images.

### YOLOv8

YOLOv8 was proposed by Jocher et al. (25); this algorithm optimized the design of the CNN layer called Cross-Stage Partial DarkNet (CSPDarkNet) to be more collegiate than YOLOv5, while the neck side uses the Path Aggregation Feature Pyramid Network (PAFPN) to improve feature fusion; the head side finally uses the anchor-free method. Wehbe et al. (25) used an anchor-free method, that is, a Pyramid Network (PAFPN), to improve feature fusion, and for the head part, an anchor-free method (25). Wehbe et al. (26) used publicly available data to evaluate the performance of YOLOv8 and developed a TNM classification model for tumor, lymph node, and metastasis (TNM) stage classification, which was found to be 98% accurate. Yao et al. (27) developed a YOLO-based framework with an attention gather and distribute strategy (GDB) to combine high-level and low-level semantic features and spatial details, concluding that the model has good prediction results on different datasets.

### YOLOv9

YOLOv9, proposed by Wang et al. (28), is an algorithm that adopts the concept of programmable gradient information (PGI) to target the various variations required by multiple objectives and introduces the Generalized Efficient Layer Aggregation Network (GELAN) to improve the efficiency of parameter usage set computations, whereas Chien et al. utilized the YOLOv9 algorithm. The network (GELAN) was introduced to improve the efficiency of parameter use set computations (28). Chien et al. (29) used the YOLOv9 algorithm for fracture detection and argued that it is effective in improving the performance of the current state-of-the-art model. Aziz and Saputri (30) proposed a method using the YOLOv9 model for the detection of skin lesions and concluded that the method can detect common skin diseases. Gui et al. (31) proposed a Feature-Based YOLOv9 (FS-YOLOv9) model for the customization of breast cancer detection and concluded that this model can achieve superior performance compared to the original YOLOv9 model.

### YOLOv10 and YOLOv11

YOLOv10, proposed by Wang et al. (32), is involved in the training for removing NMS, and the approach is effective in terms of increasing the processing speed as well as improving the competitiveness of the model. Ahmed and Manaf (33) utilized a variety of YOLOv10 for evaluation and applied it in X-rays to detect wrist fractures in children, concluding that the model is superior to YOLOv9. Ali et al. (34) proposed the use of YOLOv10 for skin cancer detection and concluded that the accuracy of the model could be improved by using the model with preprocessing and enhancement methods. YOLOv11, proposed by Jocher and Qui (35), is an algorithm that has also been optimized in the architecture of the trunk and neck to enhance feature extraction, and YOLOv11 could be deployed in environments such as edge devices and cloud-based platforms.

### Model evaluation

In this study, the performance and training progress of the model are measured by monitoring the loss functions, metrics, and learning rate. The bounding box loss (box_loss) was used to measure the error of the bounding box and the ground truth box. Different loss functions are often used to calculate similarity to improve the accuracy of object localization. The Classification Loss (CLS_Loss) evaluates the accuracy of the model in predicting the object classes. Typically, it is optimized using Cross-Entropy Loss to ensure the reliability of the object classification. The Distribution Focal Loss (DFL_Loss) further improves the accuracy of bounding box prediction; thus, the model can more accurately return the location of objects. When evaluating model performance, the Mean Average Precision (mAP) metric is often used to measure accuracy. mAP50 denotes the average precision (AP) of the IoU threshold of 0.50, particularly for large objects. mAP50-95 is the average mAP of all categories between IoU thresholds of 50 and 95% (in 5% intervals), and the IoU formula is shown in Equation (1). Precision measures the number of objects predicted by the model that are real objects, as shown in Equation (2), whereas Recall calculates the proportion of the actual number of objects successfully detected by the model, as shown in Equation (3). Therefore, YOLO’s evaluation method covers a wide range of factors, such as object localization, category classification, learning rate adjustment, and model accuracy, to ensure the stability and performance of the model in different scenarios by analyzing these key indicators. By analyzing these key indicators, we can effectively compare the applicability of different YOLO versions in detecting objects in CT images of lung cancer, which further enhances the value of computer-assisted diagnosis (CAD) in the field of medical imaging.


(1) $$IoU=\frac{\mathrm{Area}\;\mathrm{of}\;\mathrm{Overlap}}{\mathrm{Area}\;\mathrm{of}\;\mathrm{Union}}$$

(2) $$\mathrm{Precision}=\frac{TP}{TP+FP}$$

(3) $$\mathrm{Recall}=\frac{TP}{TP+FN}$$

where TP is a correctly detected object, FP is the background area that is incorrectly detected as an object, and FN is an object that is not detected.

## Experimental design and performance evaluation

### Dataset collection and data processing

The lung-PET-CT-Dx dataset used in this study contained CT and PETCT DICOM files and XML-annotated files for Adenocarcinoma, Small Cell Carcinoma, Large Cell Carcinoma, and Squamous Cell Carcinoma (36). In the data preprocessing part of the data, the images downloaded from the public data were in the DICOM format; therefore, in this study, the DICOM suite was used to convert the DICOM format images into JPG format. During the conversion process, the images were adjusted to a lung window with a window center of −400 and a window width of 1,600, according to the common practice of clinicians in lung image analysis, followed by numerical conversion according to Equations (4–7). The pixel values of the original DICOM images were mapped to a range from 0 to 255, and the processed images were saved in JPG format. In terms of annotation, the XML annotation of the YOLO training dataset was not available. This study used Python programming language to convert the XML file to a TXT file so that YOLO annotations could be read. The total number of DICOM images in the dataset was 30,738, and PET-CT images were removed for this study. The final number of data strokes is 20,381. To ensure data feasibility, the dataset was split into 16,304 images for the training set and 4,075 images for the testing set. The Dataset Demographics are shown in Table 1. There were 9,570 males (58.70%) and 6,734 females (41.30%) in the training dataset. For the testing dataset, there were 9,570 male data points, accounting for 58.53% of the total, and 6,734 female datapoints, accounting for 41.47%, respectively. In terms of age distribution, the mean age of males in the training dataset was 62.2 years old with a standard deviation range of 53.1 to 71.4 years, while the mean age of females was 62.6 years old with a standard deviation range of 52.5 to 72.7 years old. In the empirical dataset, the mean age of males was 62.4 years with a standard deviation range of 53.0–71.9 years, while the mean age of females was 62.7 years with a standard deviation range of 52.7–72.8 years.


(4) $$R_{p}=\left(I_{p}*R_{s}\right)+R_{i}$$

(5) $$W_{\text{max}}=W_{c}+\frac{W_{w}}{2}$$

(6) $$W_{\text{min}}=W_{c}-\frac{W_{w}}{2}$$

(7) $$\hat{R_{p}}=\frac{R_{p}-W_{\text{min}}}{\left(W_{\text{max}}-W_{\text{min}}\right)*255}$$

**Table 1:** Dataset demographics.

Demographic information	Training dataset		Testing dataset	
	Male	Female	M	F
Sex	9,570 (58.70%)	6,734 (41.30%)	2,385 (58.53%)	1,690 (41.47%)
Age	62.2 (53.1–71.4)	62.6 (52.5–72.7)	62.4 (53.0–71.9)	62.7 (52.7–72.8)

where $R_{p}$ represents the original DICOM image, $I_{p}$ denotes the pixel value of the current DICOM image, $R_{s}$ is the Rescale Slope value, $R_{i}$ is the Rescale Intercept value, $W_{\text{min}}$ is the minimum pixel value after conversion, $W_{\text{max}}$ is the maximum pixel value after conversion, $W_{c}$ refers to the Window Center value for conversion, $W_{w}$ represents the Window Width value for conversion, and $\hat{R_{p}}$ is the intensity-normalized value after standardization.

### Experimental environment and parameter settings

All YOLO versions were based on an ultralytics package and trained on an NVIDIA RTX 4090 GPU. The programming language in this model is Python 3.10. All model parameter settings are shown in Table 2. The performance of the model versions is shown in Table 3.

**Table 2:** Model parameter settings.

Parameters	Values
Epochs	100
Image_size	512
Batch_size	16
Optimizer	Adam
Pretrained	False

**Table 3:** Performance of the model versions.

Parameters	Size	mAPval50-95	Params	FLOPs
YOLOv5	640	49.0	25.1	64.2
YOLOv8	640	50.2	25.9	78.9
YOLOv9	640	51.4	20.1	76.8
YOLOv10	640	51.1	15.4	59.1
YOLOv11	640	51.5	20.1	68.0

### Performance evaluation

**Figure 2:**
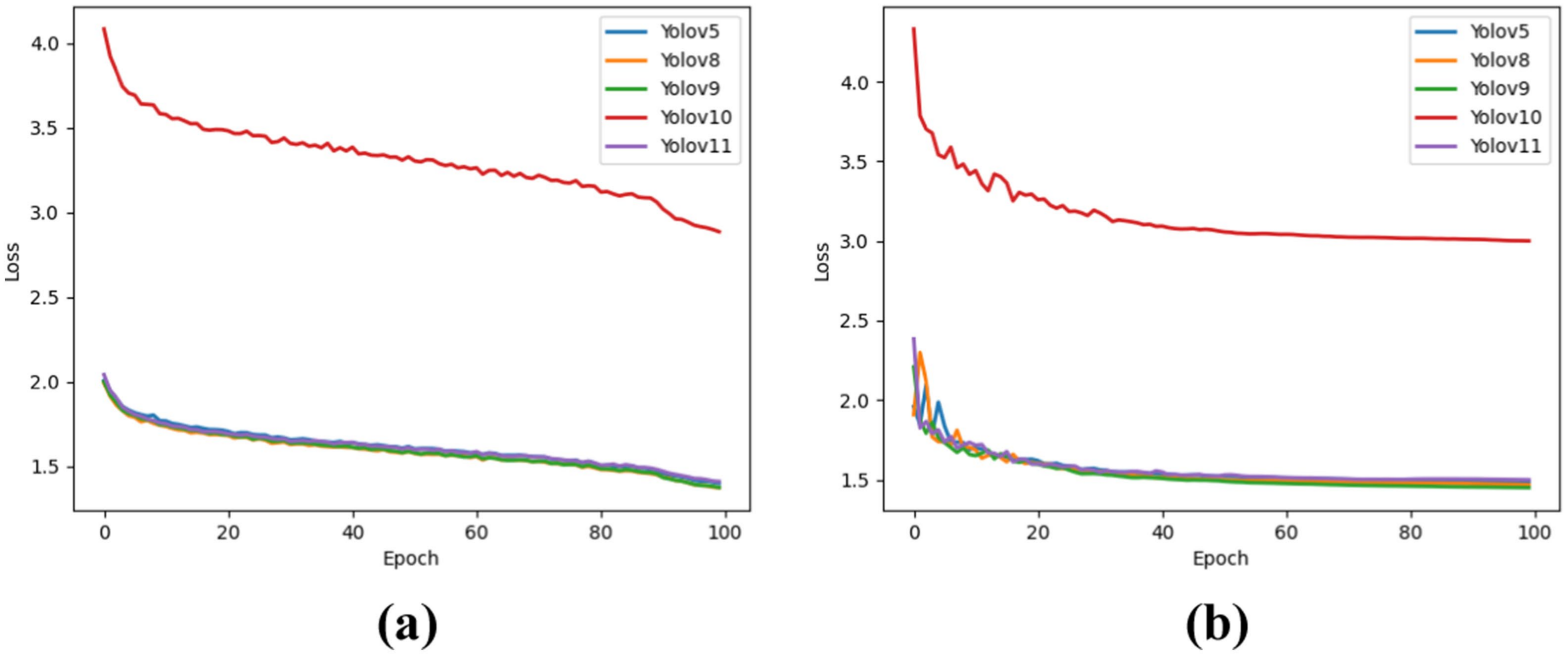
Box loss for each model in the training set test set. **(a)** For training dataset. **(b)** For testing dataset.

The results exhibit the Box_Loss of each YOLO version for both the training and test datasets. It is observed that YOLOv10 performs poorly in lung cancer detection, as indicated by its higher loss value compared to the other models. The performances of the other models were comparable, demonstrating better learning rates and more accurate target localization. YOLOv8 results revealed that the best Box_Loss for the training dataset was 1.371, followed by YOLOv9 at 1.372. For the test dataset, YOLOv9 performed the best, with a Box Loss of 1.45, followed by YOLOv8 at 1.47. Figure 2a illustrates Box_Loss for the training dataset, and Figure 2b presents Box_Loss for the test dataset.

The results show the CLS_Loss of each YOLO version for both the training and test datasets. It is observed that YOLOv10 performs poorly in lung cancer detection, as indicated by its higher loss value compared to the other models. The performance of the other models was comparable, demonstrating better learning rates and more accurate target localization. YOLOv8 results revealed that Box_Loss exhibited the best performance on the training dataset at 1.371, followed by YOLOv9 at 1.372. For the test dataset, YOLOv9 demonstrated the best performance, with a box loss of 1.45, followed by YOLOv8 at 1.47. Figure 2a shows Box_Loss for the training dataset, and Figure 2b shows Box_Loss for the test dataset.

**Figure 3:**
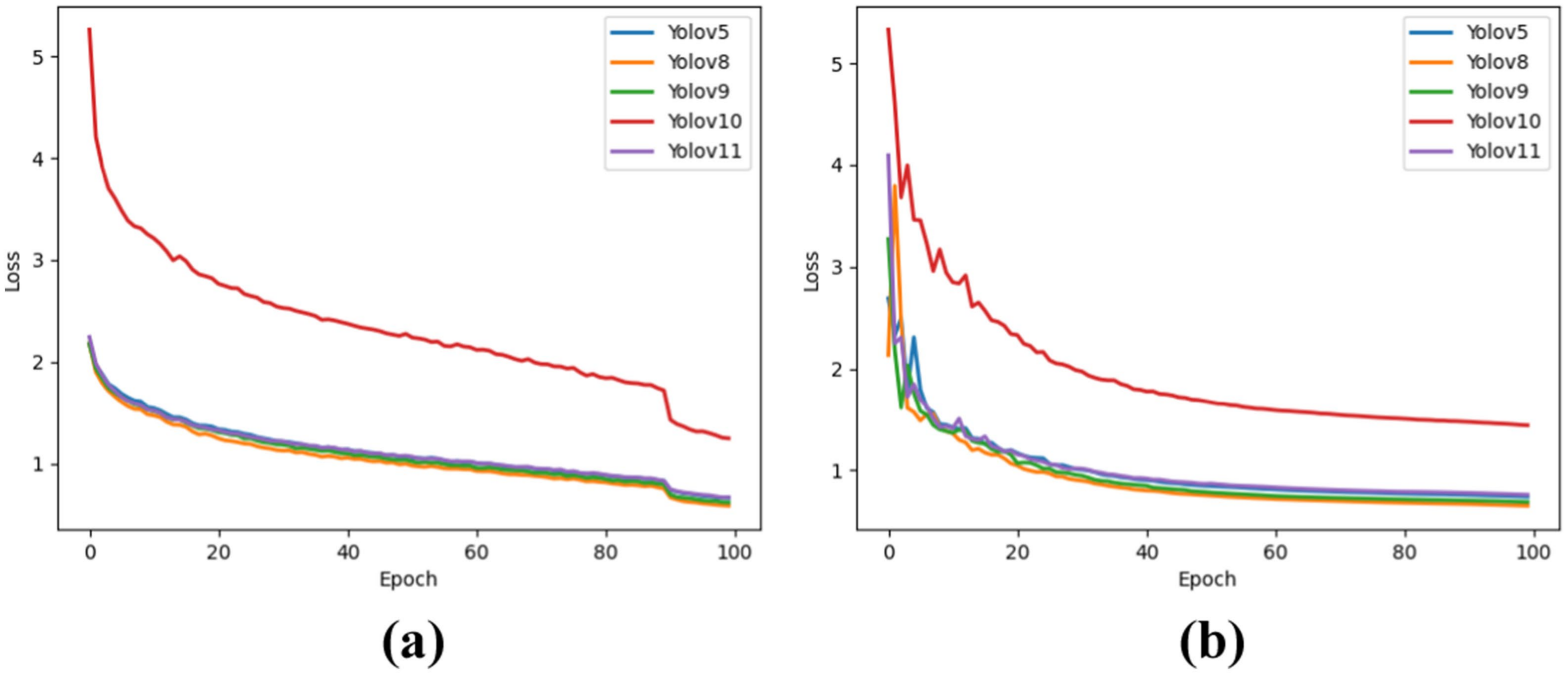
CLS loss for each model in the training and test sets. **(a)** For training dataset. **(b)** For testing dataset.

The results show the CLS_Loss values of each YOLO version for both training and test datasets. YOLOv10 performed poorly in lung cancer detection, as indicated by its higher loss value compared to the other models. The performances of the other models were comparable, demonstrating better learning capability and more accurate target localization. YOLOv8 achieved the best CLS_Loss during training at 0.59, followed by YOLOv9 at 0.62. For the test dataset, YOLOv8 again performed the best, with CLS_Loss = 0.65, followed by YOLOv9 at 0.69. Figure 3a shows CLS_Loss for the training dataset, and Figure 3b shows CLS_Loss for the test dataset.

**Figure 4:**
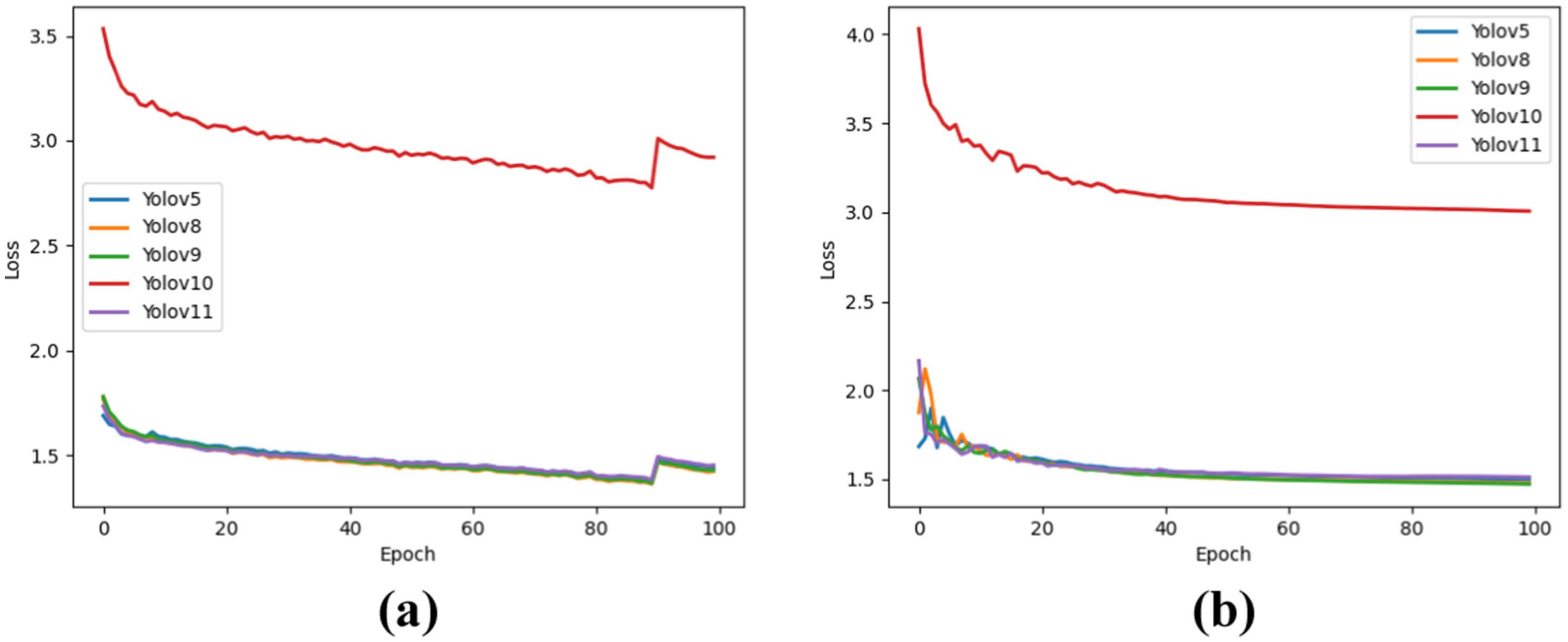
DFL_Loss for each model in the training and test sets. **(a)** For training dataset. **(b)** For testing dataset.

The results show the DFL_Loss of each YOLO version for both the training and test datasets. It was observed that YOLOv10 performed poorly in lung cancer detection, as indicated by its higher loss value compared to the other models. The performances of the other models were comparable, demonstrating better learning capability and more accurate target localization. YOLOv8 achieved the best DFL_Loss in the training dataset at 1.42, followed by YOLOv9 at 1.43. For the test dataset, YOLOv9 performs best with a DFL_Loss of 1.47, followed by YOLOv8 at 1.48. Figure 4a illustrates the DFL_Loss for the training dataset, whereas Figure 4b presents the DFL_Loss for the test dataset.

**Figure 5:**
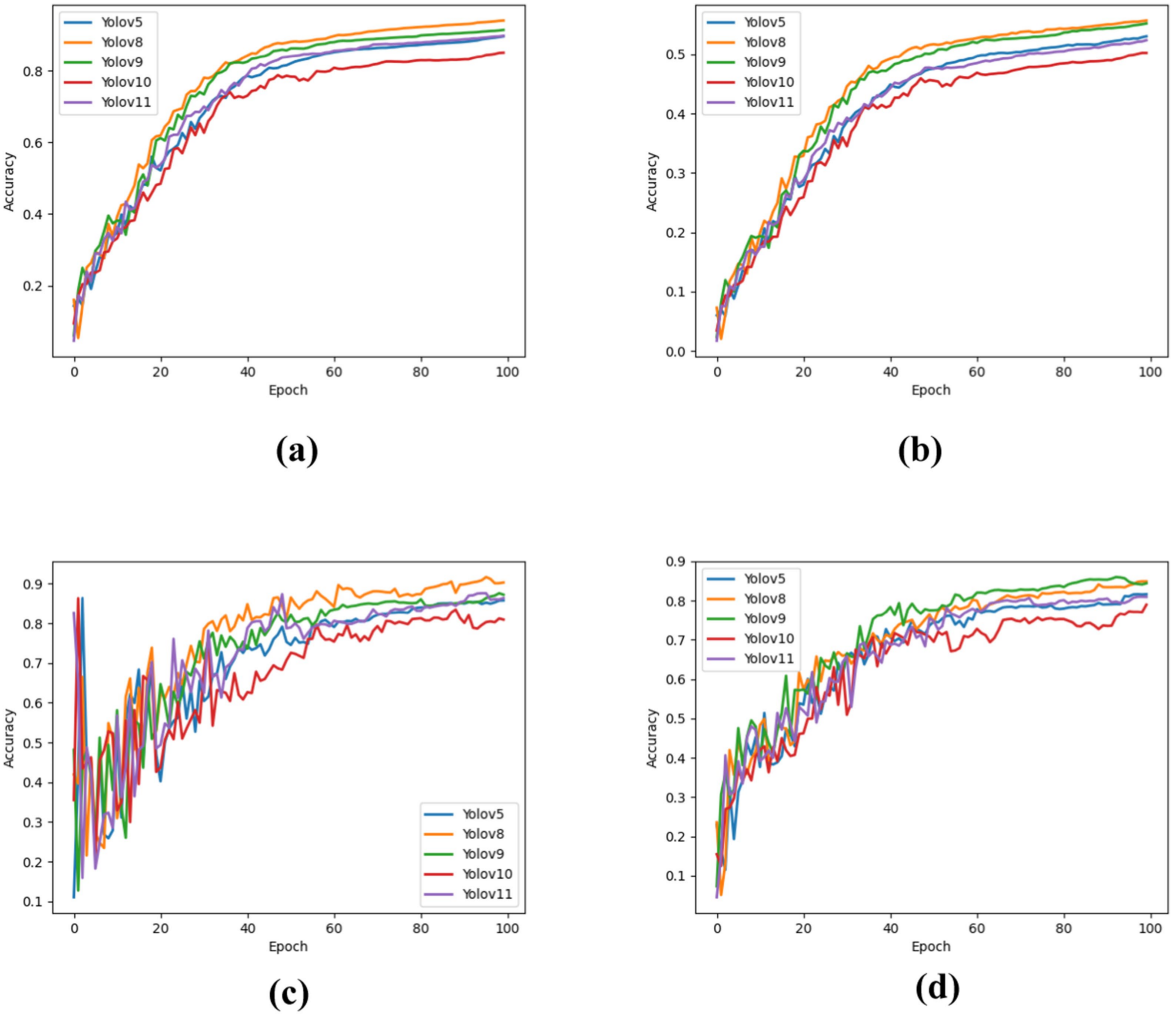
Model effectiveness evaluation. **(a)** mAP50, **(b)** mAP50-95, **(c)** Precision, and **(d)** Recall.

**Figure 6:**
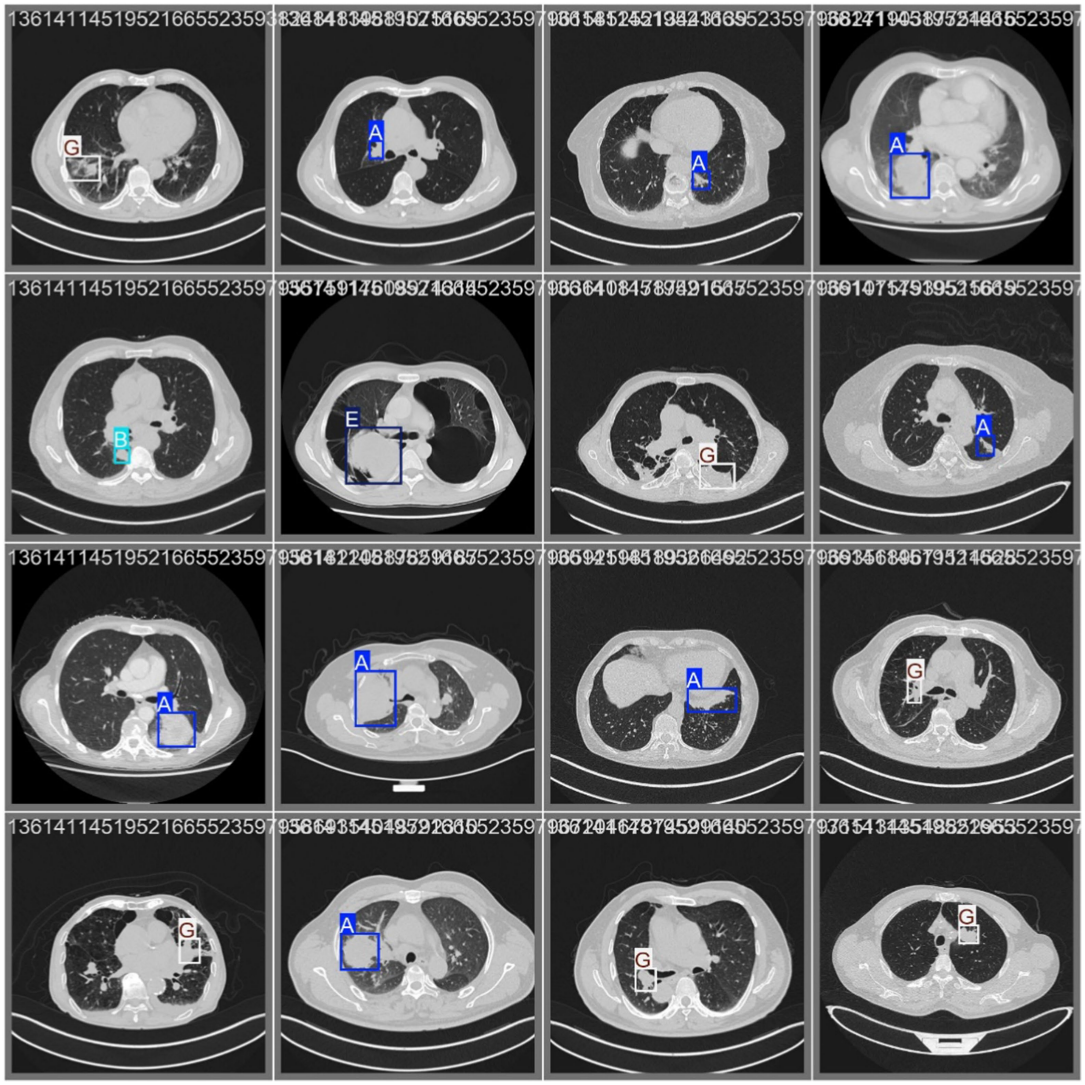
YOLOv8 model prediction results.

The results show the detection performance of each YOLO version on the test set. Figure 5 illustrates the evaluation metrics, including mAP50, mAP50-95, Precision, and Recall. Among these, Figure 5a shows that YOLOv8 achieves the highest mAP50 score (94.01%), followed by YOLOv9, YOLOv11, and YOLOv5, while YOLOv10 performs the worst. Similarly, in Figure 5b, YOLOv8 again demonstrates the best performance with an mAP50-95 score of 55.70%, followed by YOLOv9, YOLOv5, YOLOv11, and YOLOv10, which are the weakest models. For the Precision metric shown in Figure 5c, YOLOv8 achieved the highest accuracy of 90.32%, followed by YOLOv9, YOLOv11, and YOLOv5, with YOLOv10 ranking the lowest. The Recall metric, as presented in Figure 5d, also indicates that YOLOv8 leads with 84.91%, followed by YOLOv9, YOLOv5, and YOLOv11, whereas YOLOv10 again performs the worst. Overall, YOLOv8 consistently outperformed the other models across all evaluation metrics, whereas YOLOv10 showed the weakest performance. Figure 6 presents the prediction results of the YOLOv8 model on the test dataset, highlighting its efficacy in lung cancer detection. In the figure, A corresponds to Adenocarcinoma, B to Small Cell Carcinoma, E to Large Cell Carcinoma, and G to Squamous Cell Carcinoma.

Table 4 presents the mean and standard deviation of each model for the different indicators in this study. From the results, it can be seen that the box_loss, cls_loss, and dfl_loss indicators of YOLOv10 in the Training and Tesing phases show more significant differences in the values compared with the other models, which reveals greater fluctuations, probably reflecting the model’s loss function convergence or the higher error in the training process. YOLOv8 demonstrated better performance in terms of Precision, Recall, mAP50, and mAP50-95 compared to other models in terms of the key performance indicators, which indicates that the model has a stronger advantage in terms of the accuracy of object detection and the overall generalization ability. These results may be related to YOLOv8’s architecture optimization, feature extraction capability, and better anchor-free design, which allows it to maintain stable detection accuracy under different IoU thresholds.

**Table 4:** Statistical results of indicators of models.

Indicators	YOLO5	YOLO8	YOLO9	YOLO10	YOLO11
Training_box_loss	1.6 (1.5–1.7)	1.6 (1.5–1.7)	1.6 (1.5–1.7)	3.3 (3.1–3.5)	1.6 (1.5–1.7)
Training_cls_loss	1.1 (0.8–1.4)	1 (0.7–1.4)	1.1 (0.8–1.4)	2.3 (1.7–3)	1.1 (0.8–1.4)
Training_dfl_loss	1.5 (1.4–1.5)	1.5 (1.4–1.5)	1.5 (1.4–1.5)	3 (2.8–3.1)	1.5 (1.4–1.5)
Testing_box_loss	1.6 (1.5–1.7)	1.6 (1.4–1.7)	1.5 (1.4–1.6)	3.2 (2.9–3.4)	1.6 (1.5–1.7)
Testing_cls_loss	1 (0.6–1.4)	0.9 (0.5–1.3)	0.9 (0.6–1.3)	2 (1.3–2.7)	1 (0.6–1.5)
Testing_dfl_loss	1.6 (1.5–1.6)	1.6 (1.5–1.6)	1.5 (1.5–1.6)	3.1 (3–3.3)	1.6 (1.5–1.6)
Precision	0.7 (0.5–0.9)	0.8 (0.6–0.9)	0.7 (0.6–0.9)	0.7 (0.5–0.8)	0.7 (0.5–0.9)
Recall	0.7 (0.5–0.8)	0.7 (0.5–0.9)	0.7 (0.5–0.9)	0.6 (0.5–0.8)	0.7 (0.5–0.8)
mAP50	0.7 (0.5–0.9)	0.8 (0.5–1)	0.7 (0.5–1)	0.7 (0.5–0.9)	0.7 (0.5–0.9)
mAP50-95	0.4 (0.3–0.5)	0.4 (0.3–0.6)	0.4 (0.3–0.6)	0.4 (0.3–0.5)	0.4 (0.3–0.5)

Table 5 shows a comparison of the performance of the models using the paired t-test and the statistical analysis results with the best-performing YOLOv8 model. Through this test, we obtained *p*-values that showed statistically significant differences between the models and YOLOv8 for the mAP50, mAP50-95, Precision, and Recall metrics, with p-values less than 0.001 indicating that these differences were statistically significant.

**Table 5:** Statistical Significance of Indicators.

Models	mAP50	mAP50-95	Precision	Recall
YOLOv5	<0.001	<0.001	<0.001	<0.001
YOLOv9	<0.001	<0.001	<0.001	<0.001
YOLOv10	<0.001	<0.001	<0.001	<0.001
YOLOv11	<0.001	<0.001	<0.001	<0.001

## Discussion

In this study, YOLOv5, YOLOv8, YOLOv9, YOLOv10, and YOLOv11 were comprehensively compared and analyzed for object-detection tasks in lung cancer CT images. The experimental results indicate that YOLOv8 outperforms the other versions in terms of mAP50, mAP50-95, Precision, and Recall metrics, demonstrating the highest detection performance. In conclusion, YOLOv8 is the most promising model for lung cancer object detection, tumor localization, and type classification, and it provides more accurate auxiliary diagnostics for medical image analysis.

In terms of computational resource requirements, YOLOv8 has a higher number of parameters (params) and floating-point operations (FLOPs) than the other YOLO versions. These results suggest the increased complexity and computational demands of feature extraction and deep learning processes while also reinforcing their advantages in high-accuracy application scenarios.

This study excluded PET/CT datasets and focused on CT image analyses. Specific assumptions regarding artifacts have not been made; therefore, the model may not be able to completely eliminate the influence of artifacts on diagnosis. In practice, the results of the model should be used in conjunction with the clinician’s interpretation to ensure diagnostic accuracy. In addition, because the performance of the model may be affected by the type of equipment and data source, continuous data updates and fine-tuning are required in the future to enhance its adaptability and ensure its stability in different clinical settings.

The CT-based object detection model for lung cancer can help medical professionals accurately locate lung cancer lesions, thereby enhancing diagnostic precision. In predicting lung cancer types, the model achieves an accuracy of 90.32%, demonstrating its feasibility and practical application value. The integration of this technology can support clinicians in making early diagnoses and developing timely and appropriate treatment plans, ultimately contributing to improved patient survival rates and prognoses.

This study was conducted using publicly available datasets. Future research should focus on validating the model using clinical data obtained from hospitals. The inclusion of data from diverse ethnic groups enhances the credibility and generalizability of the model. In future research, this study will incorporate additional deep-learning models to further evaluate and enhance the effectiveness of predictive modeling. Advanced image recognition techniques, such as U-Net- and Transformer-based models, will be integrated for model training and comparative analysis to determine their potential in improving prediction accuracy and reliability.

## Conclusion

Early diagnosis of lung cancer and accurate identification of cancer types are crucial challenges in modern medicine. The application of artificial intelligence can significantly aid doctors in rapidly identifying both the type and location of lung cancer, representing an important breakthrough in medical diagnostics. This study compares different versions of YOLO to determine which model yields the best predictive performance. The results indicate that the YOLOv8 model outperforms the other four YOLO versions, achieving a precision of 90.32% and a recall of 84.91%. These findings demonstrate that YOLOv8 can effectively assist physicians in diagnosing lung cancer with high accuracy.

## Data Availability

The datasets presented in this study can be found in online repositories. The names of the repository/repositories and accession number(s) can be found at: https://www.cancerimagingarchive.net/collection/lung-pet-ct-dx/.

## References

[1] Chhikara BS Parang K. Global cancer statistics 2022: the trends projection analysis. Chem. Biol. Lett. (2023) 10:451–1. Available at: https://pubs.thesciencein.org/journal/index.php/cbl/article/view/451

[2] Wang BY Huang JY Cheng CY Lin CH Ko JL Liaw YP. Lung cancer and prognosis in Taiwan: a population-based cancer registry. J Thorac Oncol. (2013) 8:1128–35. doi: 10.1097/JTO.0b013e31829ceba4, PMID: 23945383

[3] Chang YJ Huang JY Lin CH Wang BY. Survival and treatment of lung cancer in Taiwan between 2010 and 2016. J Clin Med. (2021) 10:4675. doi: 10.3390/jcm10204675, PMID: 34682798 PMC8540538

[4] Kratzer TB Bandi P Freedman ND Smith RA Travis WD Jemal A. Lung cancer statistics, 2023. Cancer. (2024) 130:1330–48. doi: 10.1002/cncr.35128, PMID: 38279776

[5] Rudin CM Brambilla E Faivre-Finn C Sage J. Small-cell lung cancer. Nat Rev Dis Prim. (2021) 7:3. doi: 10.1038/s41572-020-00235-0, PMID: 33446664 PMC8177722

[6] Swensen SJ. CT screening for lung cancer. Am J Roentgenol. (2002) 179:833–6. doi: 10.2214/ajr.179.4.1790833 12239020

[7] Ambrosini V Nicolini S Caroli P Nanni C Massaro A Marzola MC. PET/CT imaging in different types of lung cancer: an overview. Eur J Radiol. (2012) 81:988–1001. doi: 10.1016/j.ejrad.2011.03.020, PMID: 21458181

[8] Jannatdoust P Valizadeh P Saeedi N Valizadeh G Salari HM Saligheh Rad H. Computer-aided detection (CADe) and segmentation methods for breast Cancer using magnetic resonance imaging (MRI). J Magn Reson Imaging. (2025). doi: 10.1002/jmri.29687, PMID: 39781684

[9] Mushtaq F Bhattacharjee S Mandia S Singh K Chouhan SS Kumar R. Artificial intelligence for computer aided detection of pneumoconiosis: a succinct review since 1974. Eng Appl Artif Intell. (2024) 133:108516. doi: 10.1016/j.engappai.2024.108516

[10] Qin ZZ Van der Walt M Moyo S Ismail F Maribe P Denkinger CM. Computer-aided detection of tuberculosis from chest radiographs in a tuberculosis prevalence survey in South Africa: external validation and modelled impacts of commercially available artificial intelligence software. Lancet Digital Health. (2024) 6:e605–13. doi: 10.1016/S2589-7500(24)00118-3, PMID: 39033067 PMC11339183

[11] Lakshmanaprabu SK Mohanty SN Shankar K Arunkumar N Ramirez G. Optimal deep learning model for classification of lung cancer on CT images. Futur Gener Comput Syst. (2019) 92:374–82. doi: 10.1016/j.future.2018.10.009

[12] Pang S Zhang Y Ding M Wang X Xie X. A deep model for lung cancer type identification by densely connected convolutional networks and adaptive boosting. IEEE Access. (2019) 8:4799–805. doi: 10.1109/ACCESS.2019.2962862, PMID: 39573497

[13] Wang S Dong L Wang X Wang X. Classification of pathological types of lung cancer from CT images by deep residual neural networks with transfer learning strategy. Open Med. (2020) 15:190–7. doi: 10.1515/med-2020-0028, PMID: 32190744 PMC7065426

[14] Gautam N Basu A Sarkar R. Lung cancer detection from thoracic CT scans using an ensemble of deep learning models. Neural Comput Applic. (2024) 36:2459–77. doi: 10.1007/s00521-023-09130-7

[15] Du L Zhang R Wang X. Overview of two-stage object detection algorithms In: Journal of physics: Conference series, vol. 1544. Bristol: IOP Publishing (2020). 012033.

[16] Ren S He K Girshes R Sun J. Faster R-CNN: towards real-time object detection with region proposal networks. IEEE Trans Pattern Anal Mach Intell. (2016) 39:1137–49. doi: 10.1109/TPAMI.2016.2577031, PMID: 27295650

[17] Dai J Li Y He K Sun J. R-fin: object detection via region-based fully convolutional networks. Adv. Neural Inform. Proces. Syst. (2016):29. doi: 10.48550/arXiv.1605.06409

[18] Cai Z. Vasconcelos N. (2018). Cascade r-canny: delving into high quality object detection. In Proceedings of the IEEE conference on computer vision and pattern recognition (pp. 6154–6162).

[19] Deng J Xuan X Wang W Li Z Yao H Wang Z. A review of research on object detection based on deep learning In: Journal of physics: Conference series, vol. 1684. Bristol: IOP Publishing (2020). 12028.

[20] Redmon J. (2016). You only look once: unified, real-time object detection. In Proceedings of the IEEE conference on computer vision and pattern recognition.

[21] Liu W. Anguelov D. Erhan D. Szegedy C. Reed S. Fu C. Y.. (2016). SSD: single shot multibox detector. In Computer vision–ECCV 2016: 14th European conference, Amsterdam. October 11–14, 2016 (pp. 21–37).

[22] Jocher G Chaurasia A Stoken A Borovec J Kwon Y Michael K. Ultralytics/yolov5: v7. 0-yolov5 sota realtime instance segmentation. Geneva: Zenodo (2022).

[23] Begum RS Devi US. Enhanced BRAIN disorder detection through YOLOV5 in medical image analysis. Machine Intell. Res. (2024) 18:906–22. Available at: https://machineintelligenceresearchs.com/index.php/mir/article/view/77

[24] Chen S Duan J Wang H Wang R Li J Qi M. Automatic detection of stroke lesion from diffusion-weighted imaging via the improved YOLOv5. Comput Biol Med. (2022) 150:106120. doi: 10.1016/j.compbiomed.2022.106120, PMID: 36179511

[25] Jocher G. Chaurasia A. Qiu J. (2023). Ultralytics YOLO (version 8.0. 0) [computer software]. Available online at: https://github.com/ultralytics/ultralytics

[26] Wehbe A Dellepiane S Minetti I. Enhanced lung Cancer detection and TNM staging using YOLOv8 and TNMClassifier: An integrated deep learning approach for CT imaging. IEEE Access. (2024) 12:141414–24. doi: 10.1109/ACCESS.2024.3462629

[27] Yao Q Zhuang D Feng Y Wang Y Liu J. Accurate detection of brain tumor lesions from medical images based on improved YOLOv8 algorithm. IEEE Access. (2024) 12:144260–79. doi: 10.1109/ACCESS.2024.3472039

[28] Wang CY Yeh IH Mark Liao HY. Yolov9: learning what you want to learn using programmable gradient information In: European conference on computer vision. Cham: Springer (2025). 1–21.

[29] Chien CT Ju RY Chou KY Chiang JS. YOLOv9 for fracture detection in pediatric wrist trauma X-ray images. Electron Lett. (2024) 60:e13248. doi: 10.1049/ell2.13248

[30] Aziz F Saputri DUE. Efficient skin lesion detection using YOLOv9 network. J. Med. Informat. Technol. (2024):11–5. doi: 10.37034/medinftech.v2i1.30

[31] Gui H Su T Jiang X Li L Xiong L Zhou J. FS-YOLOv9: a frequency and spatial feature-based YOLOv9 for real-time breast cancer detection. Acad Radiol. (2024) 32:1228–40. doi: 10.1016/j.acra.2024.09.048 39406579

[32] Wang A Chen H Liu L Chen K Lin Z Han J. Yolov10: real-time end-to-end object detection. arXiv. (2024) doi: 10.48550/arXiv.2405.14458

[33] Ahmed A Manaf A. Pediatric wrist fracture detection in x-rays via yolov10 algorithm and dual label assignment system. arXiv. (2024). doi: 10.48550/arXiv.2407.15689

[34] Ali B. S. Nasir H. Khan A. Ashraf M. Akbar S. M. (2024) A machine learning-based model for the detection of skin Cancer using YOLOv10. In 2024 IEEE 8th international conference on signal and image processing applications (ICSIPA) (pp. 1–6).

[35] Jocher G. Qiu J. (2024). Ultralytics YOLO11 (version 11.0. 0) [computer software]. Available online at: https://github.com/ultralytics/ultralytics

[36] Li P Wang S Li T Lu J HuangFu Y Wang D. A large-scale CT and PET/CT dataset for lung Cancer diagnosis (lung-PET-CT-dx) [data set]. Cancer Imag. Arch. (2020). doi: 10.7937/TCIA.2020.NNC2-0461

